# A Manual of Chemistry

**Published:** 1900-03

**Authors:** 


**A Manual of Chemistry.** By ARTHUR P. LUFF, M.D., and FREDERIC JAMES M. PAGE, B.Sc. Pp. xvi., 541. London: Cassell & Company, Limited. 1900.

This book is incomparably better than its predecessor, known as Luff's *Chemistry*. In the preface to the previous work it was suggested, as a recommendation, that the wants of a medical student could be best gauged by one who had been through the courses qualifying for medicine. This may be so, but something more was needed, and the author is to be congratulated on having now secured the collaboration of a chemist, with the result that the inaccuracies which disfigured the original volume have been removed. The principal on which Luff's *Chemistry* appears to have been written was to start with certain preconceived theories; and if the facts did not fit them, so much the worse for the facts. They were simply altered so as to make them fit; thus the formula for nitric oxide was written $N_2O_2$ instead of N O, in order to make nitrogen a triad.

In the present volume the facts—except for a few misprints such as $\left\{\begin{smallmatrix}Ca\,Cl\\Ca\,O\,Cl\end{smallmatrix}\right.$ instead of $Ca\left\{\begin{smallmatrix}Cl\\O\,Cl\end{smallmatrix}\right.$, p. 139—are stated correctly, and those of the old theories which were based on false or distorted facts are discarded. The use of rational formulæ has now also rendered the description of organic chemistry readable and intelligible.

The improvement is so great that whereas the writer of this review felt it necessary to warn his students against using the original edition, he will be quite content to see the present one in their hands.

